# Histone Demethylases Coordinate the Antagonistic Interaction Between Abscisic Acid and Brassinosteroid Signaling in *Arabidopsis*

**DOI:** 10.3389/fpls.2020.596835

**Published:** 2020-11-25

**Authors:** Jinfeng Wu, Mingli Yan, Dawei Zhang, Dinggang Zhou, Nobutoshi Yamaguchi, Toshiro Ito

**Affiliations:** ^1^School of Life Sciences, Hunan University of Science and Technology, Xiangtan, China; ^2^Division of Biological Science, Nara Institute of Science and Technology, Ikoma, Japan; ^3^Hunan Key Laboratory of Economic Crops Genetic Improvement and Integrated Utilization, Hunan University of Science and Technology, Xiangtan, China; ^4^Precursory Research for Embryonic Science and Technology, Japan Science and Technology Agency, Kawaguchi-shi, Japan

**Keywords:** abscisic Acid, *Arabidopsis thaliana*, BRASSINAZOLE RESISTANT1, brassinosteroids, JUMONJI C demethylases

## Abstract

Abscisic acid (ABA) interacts antagonistically with brassinosteroids (BRs) to control plant growth and development in response to stress. The response to environmental cues includes hormonal control via epigenetic regulation of gene expression. However, the details of the ABA–BR crosstalk remain largely unknown. Here, we show that JUMONJI-C domain containing histone demethylases (JMJs) coordinate the antagonistic interaction between ABA and BR signaling pathways during the post-germination stage in *Arabidopsis*. BR blocks ABA-mediated seedling arrest through repression of *JMJ30*. JMJs remove the repressive histone marks from the *BRASSINAZOLE RESISTANT1* (*BZR1*) locus for its activation to balance ABA and BR signaling pathways. JMJs and BZR1 co-regulate genes encoding three membrane proteins, a regulator of vacuole morphology, and two lipid-transfer proteins, each of which play a different role in transport. BZR1 also regulates stimuli-related target genes in a JMJ-independent pathway. Our findings suggest that the histone demethylases integrate ABA and BR signals, leading to changes in growth program after germination.

## Introduction

Plants have the ability to respond to various environmental stimuli over the course of their development (Blatt et al., 2017; Bechtold and Field, 2018). These responses are characterized by extensive phenotypic plasticity, and entail a balance between stress response and growth (Foyer et al., 2007; Sanchez-Bel et al., 2018). In this flexible and reversible balance, plants minimize the damage caused by stress and maximize growth. However, we do not fully understand how plants control this intricate balance.

The phytohormone abscisic acid (ABA) coordinates stress responses and inhibits plant growth (Yamaguchi-Shinozaki and Shinozaki, 2006; Cutler et al., 2010; Finkelstein, 2013). In the absence of ABA, type 2C protein phosphatases (PP2Cs) physically interact with SNF1-RELATED PROTEIN KINASE 2s (SnRK2s) to inhibit protein phosphorylation (Hrabak et al., 2003). ABA binds to receptors such as PYRABACTIN RESISTANCE1 (PYR1), PYR1-LIKE (PYL), and REGULATORY COMPONENTS OF ABA RECEPTOR (RCAR). This triggers conformational changes that enable protein-protein interaction between PYRs and PP2Cs, such as ABSCISIC ACID INSENSITIVE 1 (ABI1), ABI2, and HYPERSENSITIVE TO ABA1 (HAB1; Park et al., 2009; Cutler et al., 2010). The conformational changes release SnRK2s from inhibition by PP2Cs. The SnRK2s activate downstream transcription factors such as the bZIP-type transcription factor ABSCISIC ACID INSENSITIVE5 (ABI5), the AP2-type protein ABI4, the B3-type protein ABI3, and ABA-responsive elements-binding factors (ABFs), through phosphorylation (Lopez-Molina et al., 2002; Nakashima et al., 2009). These transcription factors regulate the expression of ABA-responsive genes including stress response and growth-inhibition targets (Suzuki et al., 2003; Nakabayashi et al., 2005).

Another class of phytohormones, BRs, promote plant growth and development. The plasma membrane-localized serine/threonine kinase BRASSINOSTEROID-INSENSITIVE1 (BRI1) recognizes BRs (Clouse, 2011; Zhang et al., 2018). BRI1-BR binding triggers conformational changes that enable heterodimer formation between BRI1 and BRASSINOSTEROID INSENSITIVE-ASSOCIATED RECEPTOR KINASE 1 (BAK1; Li and Chory, 1997; Nam and Li, 2002; Cano-Delgado et al., 2004; Fabregas et al., 2013). These changes initiate intracellular phosphorylation pathways: the BRI1/BAK1 heterodimer leads to promotion of the activity and stability of the plant-specific transcription factors BRASSINAZOLE RESISTANT1 (BZR1) and bri1-EMS-SUPPRESSOR1 (BES1), which lead to transcriptional activation of BR-responsive genes for plant growth and development (Yin et al., 2002; Ibanez et al., 2018; Tian et al., 2018).

Recent studies suggest that ABA and BRs antagonistically regulate environmental responses (Hu and Yu, 2014; Ryu et al., 2014; Skubacz et al., 2016). Brassinosteroid biosynthetic and signaling mutants are hypersensitive to exogenous ABA; they are hyposensitive to ABA when these BR biosynthetic and signaling genes are overexpressed (Clouse et al., 1996). BIN2 is a key module that controls BR–ABA crosstalk (Gruszka, 2013). BIN2 stabilizes ABI5 through phosphorylation in the presence of ABA (Hu and Yu, 2014). SnRK2.2, SnRK2.3, and SnRK2.6 are also phosphorylation substrates of BIN2 (Cai et al., 2014; Shang et al., 2016). In addition, BZR1 directly regulates *ABI5* expression (Yang et al., 2016). Hence, the transcriptional regulatory network underlying the ABA–BR crosstalk is at least partially characterized.

Epigenetic regulation mediates reversible changes in gene expression. The type and degree of histone modification is often affected by environmental stimuli (Kim et al., 2008; Luo et al., 2012). H3 lysine 27 trimethylation (H3K27me3) is associated with a repressed chromatin state and gene silencing (Zhang et al., 2007; Lafos et al., 2011; Wu et al., 2012; Sun et al., 2014), and is responsive to environmental changes (Liu et al., 2014; Qian et al., 2018). H3 lysine 27 trimethylation demethylases have important roles in regulating these dynamics (Tsukada et al., 2006; Yamane et al., 2006). EARLY FLOWERING 6 (ELF6), RELATIVE OF EARLY FLOWERING 6 (REF6), JUMONJI C DOMAIN-CONTAINING PROTEIN 13 (JMJ13), JMJ30, and JMJ32 function as H3K27me3 demethylases in *Arabidopsis* (Lu et al., 2011; Gan et al., 2014; Zheng et al., 2019). In response to ABA, ABI3-mediated JMJ30 activation accelerates *SnRK2.8* expression through H3K27me3 demethylation (Wu et al., 2019a, b). ELF6, REF6, and BES1 form a complex in response to BRs, and control genes downstream of BES1 through H3 lysine9 trimethylation (H3K9me3; Yu et al., 2008). However, the role of H3K27me3 demethylases on the crosstalk between ABA and BR signaling pathways remains unclear.

Here, we provide insights into the role of histone demethylases in the antagonistic interaction between ABA and BRs during post-germination stage. BR blocks ABA-mediated seedling arrest through *JMJ30* repression. JMJ30 and JMJ32 remove H3K27me3 marks from the *BZR1* locus to activate *BZR1* expression. Finally, we identified genes that were shared and unique targets of JMJ and BZR1; these were involved in transport and stress responses. Our study reveals a shared regulatory network between ABA and BR that acts via JMJ-mediated histone modification and a BR-signaling master regulator.

## Materials and Methods

### Plant Materials and Plant Growth

In this study, the *Arabidopsis thaliana* Columbia (Col-0) was used and the mutants are in the Col-0 background. The *jmj30-2 jmj32-1* mutant, *pJMJ30:JMJ30-GUS*, and *pJMJ30:JMJ30-HA* have been described previously (Gan et al., 2014). Seeds were stratified for 3 days at 4°C in darkness. The plants were grown in a growth chamber at 22°C under continuous light.

### Plasmid Construction and Plant Transformation

To generate a *BZR1* overexpression construct, the full-length coding sequences (CDS) were inserted into the pENTR/D-TOPO vector (Thermo Fisher Scientific) using the primers, BZR1–cds_FW and BZR1–cds_RV. Then, the *BZR1* CDS-containing vector was recombined into pB2GW7.0 using LR Clonase II (Thermo Fisher Scientific). The primers used in cloning are listed in Supplementary Table 1.

Transgenic lines were generated by floral dipping method through *Agrobacterium tumefaciens* infection (GV3101; Clough and Bent, 1998). More than 30 *BZR1* overexpression lines were identified. Eight lines were examined to detect expression levels of *BZR1*. Two representative lines were used for further phenotypic analysis.

### Phenotypic and Statistical Analyses

Half-strength Murashige and Skoog (MS) plates without sucrose were prepared and seeds were surface-sterilized as described previously (Wu et al., 2019a). To prepare ABA-containing plates, 0.3 or 0.4 μM ABA were added to half-strength MS medium after sterilization in an autoclave (Sigma-Aldrich; 14375-45-2). For ABA- and BR-containing 1/2 MS plates, the 0.4 μM ABA-containing 1/2 MS medium was supplemented with 10 or 20 nM Brassinolide (Furashino; lot no.A122101). Solvent alone was used as a negative control. Bleach-sterilized seeds were sown on plates using a pipette under a clean bench. These plates were placed at 4°C for 3 days to stratify seeds before being transferred to a growth chamber at 22°C under continuous light. The seed germination ratios were investigated after 12, 24, 36, 48, 60, and 72 h of incubation. The seedling establishment ratios of WT and *jmj30-2 jmj32-1* were counted on the seventh day. The mean and SE were determined from three technical replicates (3 plates containing 36 plants) from three independent experiments. The photos were taken on a DSC-TX30 camera (SONY). Statistically significant differences in seed germination and seedling establishment were tested using a Chi-squared test.

### BR Treatment

For reverse transcription quantitative polymerase chain reaction (RT-qPCR) and GUS staining analyses, 36-h-old seedlings on 1/2 MS plates were immersed in 1 μM BR (Wang et al., 2018a). For RT-qPCR, the seedlings were harvested at 0, 3, and 5 h after treatment. For GUS staining, seedlings were treated for 5 h.

### GUS Staining

GUS staining was performed as previously described with minor modifications (Sun et al., 2009). Tissues were fixed in 90% cold acetone for 15 min, rinsed with sterilized ddH_2_O and GUS staining solution without X-Gluc, and incubated with GUS staining solution without 2 mM X-Gluc [50 mM NaPO_4_, 0.5 mM K_3_Fe(CN)_6_, 0.5 mM K_4_Fe(CN)_6_ at pH 7.2]. *pJMJ30:JMJ30-GUS* and *pJMJ32:JMJ32-GUS* were stained at 37°C for 2 h and overnight, respectively. The pigments from the GUS-stained tissues were removed using 70% EtOH treatment for 1 week. The tissues were photographed using an Axio Scope A1 microscope (ZEISS).

### RT-qPCR

Reverse transcription quantitative polymerase chain reaction was performed as previously described (Wu et al., 2019a). Briefly, total RNA was isolated using an RNeasy Plant Mini Kit (Qiagen). cDNA was synthesized from 2.5 μg DNase-treated RNA, using PrimeScript RT Master Mix (Takara). RT-qPCR was conducted with gene-specific primers and FastStart DNA essential DNA Green Master (Roche), using a Light Cycler 480 (Roche). *EIF4A1* (*AT3G13920*) was used as an internal control. Each experiment included four technical replicates and three biological replicates of each sample. RT-qPCR primers are described in Supplementary Table 1.

### Transcriptomic Analysis

The transcriptomic data were sourced from previously published data and public databases (Song et al., 2016; Yang et al., 2016; Wu et al., 2019a). Differentially expressed genes (DEGs) were defined by a *p* < 0.05. The Venn diagrams were made using jevenn online software.^1^ Heatmaps were made using mev software.^2^ Go analyses were performed using agriGOv2.0 (Tian et al., 2017).

### Chromatin Immunoprecipitation

Chromatin immunoprecipitation (ChIP) was performed as previously described with minor modifications (Yamaguchi et al., 2014). Briefly, 600 mg of 36-h-old seedlings were used for total chromatin extraction. The extracted chromatin was immunoprecipitated using 5 μL/sample of anti-HA antibody (Roche; 12CA5) or 5 μL/sample of anti-H3K27me3 antibody (Abcam; ab6002). After reverse-crosslinking under 65°C overnight and DNA purification, the amount of DNA was quantified by qPCR. *ACT7* (*AT5G09810*) was used as an internal control. For both H3K27me3- and HA-ChIP, all samples were normalized to the input DNA, and the mock-treated wild type was set to 1.0 to obtain the fold change for the other samples. Three independent experiments for each sample were conducted for ChIP-qPCR analysis and four technical replicates were used in each experiment. Statistically significant differences were calculated using a one-way ANOVA followed by *post hoc* Tukey’s HSD test. ChIP-qPCR primers are described in Supplementary Table 1.

## Results

### JMJ30 Expression Is Repressed by BR During the Post-germination Stage

**FIGURE 1:**
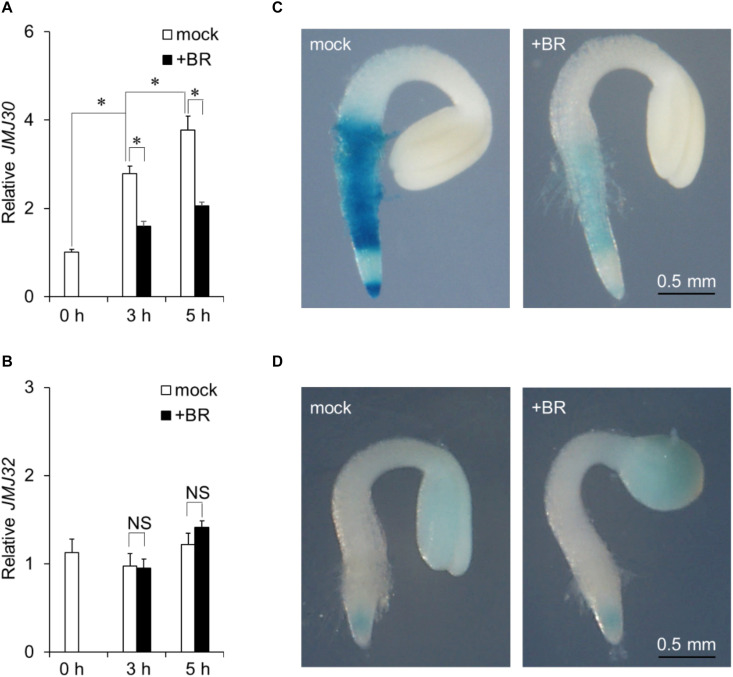
Exogenous BR application suppresses *JMJ30* upregulation during post-germination stage. **(A,B)** Expression of *JMJ30* **(A)** and *JMJ32* **(B)** based on RT-qPCR in wild-type seedlings in response to BR during post-germination stage. Data are from three independent experiments. Values represent means ± SEM. Asterisks indicate significant differences between two samples based on a two-tailed Student’s *t*-test. **(C,D)** *pJMJ30:JMJ30-GUS* **(C)** and *pJMJ32:JMJ32-GUS* **(D)** expression in wild-type background 5 h after mock or BR treatment. Scale bar = 0.5 mm.

*JMJ30* expression is induced by ABA during post-germination stage (Wu et al., 2019a). To investigate the effect of BR on *JMJ30* expression during this stage, 36-h-old wild-type plants were treated with exogenous BR. *JMJ30* was under developmental control; its expression levels increased with plant growth (mock 0 h vs. mock 3 h: *p* = 1.2 × 10^–6^; mock 3 h vs. mock 5 h: *p* = 7.0 × 10^–3^ by two-tailed Student’s *t*-test (Figure 1A; Wu et al., 2019a). This upregulation was significantly suppressed in BR-treated seedlings within 3 h after treatment, compared to that in mock-treated seedlings (mock 3 h vs. BR 3 h: *p* = 1.6 × 10^–5^ by two-tailed Student’s *t*-test; Figure 1A). The *JMJ30* expression levels also remained low at 5 h after the BR treatment (mock 5 h vs. BR 5 h: *p* = 2.5 × 10^–4^ by two-tailed Student’s *t*-test; Figure 1A). *JMJ32* expression was not affected by the presence of BR (*p* > 0.05; Figure 1B). The BR receptor-encoding *BRI1* gene was used as a positive control for BR treatment (Supplementary Figure 1).

To confirm that BR suppressed JMJ30 during post-germination stage, we examined spatial patterns of JMJ30-GUS accumulation under BR treatment in a *pJMJ30:JMJ30-GUS* line (Wu et al., 2019a). In the absence of BR, JMJ30-GUS was expressed mainly in the root meristematic and maturation zones (Figure 1C). Upon BR treatment, JMJ30-GUS levels decreased when compared with the mock-treated control (Figure 1C). We also examined JMJ32-GUS levels with and without BR treatment. Under both conditions, JMJ32-GUS accumulated in cotyledons and root tips at similar levels (Gan et al., 2014). In agreement with the RT-qPCR result, JMJ32 levels remained unchanged by BR treatment (Figure 1D). Thus, while JMJ32 maintains basal levels of stable expression, *JMJ30* expression is suppressed by BR during post-germination stage.

To test the role of JMJ on the crosstalk between ABA and BR, we examined the expression of ABA-inducible genes such as *ABI5*, *ABF2*, *ABF3*, and *ABF4* (Finkelstein and Lynch, 2000; Kang et al., 2002; Kim et al., 2004; Yoshida et al., 2010) after BR treatment. In wild-type, *jmj30-2*, and *jmj32-1* seedlings, BR inhibited the expression of all four genes; in *jmj30-2 jmj32-1* double mutants, BR repressed these genes to a lesser extent (*p* < 0.05 by one-way ANOVA test; *jmj30-1 jmj32-1* + 5h BR vs. the other samples: *p* < 0.05 by *post hoc* Tukey’s HSD test; Supplementary Figure 2). Hence, JMJ30 and JMJ32 may redundantly act as important regulators that link the BR and ABA pathways.

### JMJs Are Required for Proper BR Signaling to Inhibit ABA Pathway

Although *jmj30* and *jmj32* single mutants showed normal ABA responses, the *jmj30 jmj32* double mutants were insensitive to ABA (Wu et al., 2019a). To understand the role of JMJ30 and JMJ32 on the ABA-BR crosstalk during post-germination stage, we characterized the phenotypic differences between wild type, *jmj30-2*, *jmj32-1*, and *jmj30-2 jmj32-1* double mutants after exogenous ABA- and BR-treatments.

**FIGURE 2:**
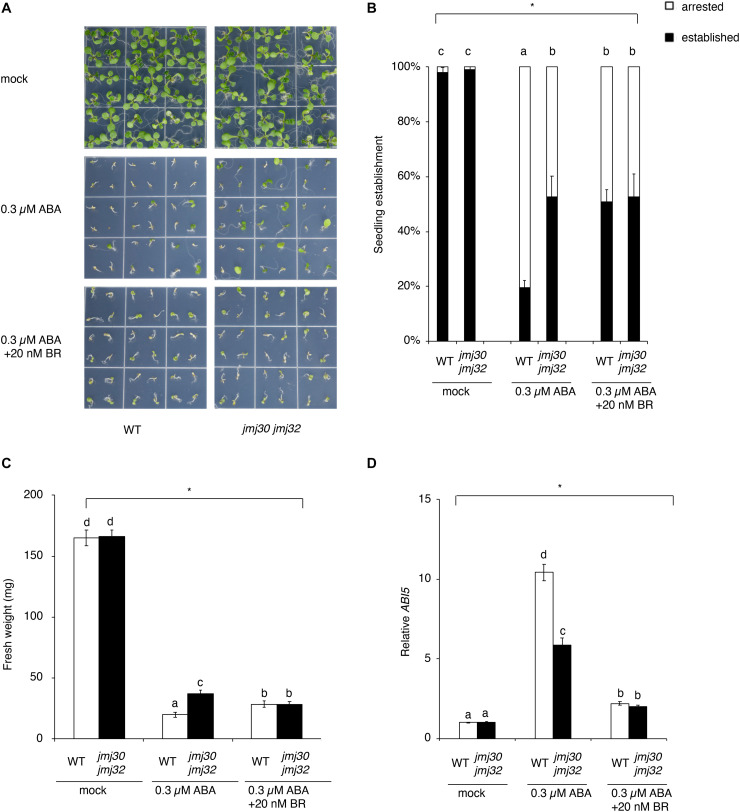
BR blocks ABA-mediated seedling arrest during post-germination stage. **(A)** Representative images of the wild type and *jmj30-2 jmj32-1* double mutants grown for 7 days on 1/2 MS medium (–sucrose) +0.3 μM ABA containing 0 and 20 μM BR. **(B)** Percentages of established seedlings in ABA-treated wild-type and *jmj30-2 jmj32-1* plants in the absence and presence of BR. Asterisks indicate significant differences based on one-way ANOVA test. *p* < 0.05. Different letters indicate significant differences, whereas the same letters indicate non-significant differences based on *post hoc* Tukey’s HSD test. *p* < 0.05. **(C)** Measurement of fresh weight in ABA-treated wild-type and *jmj30-2 jmj32-1* plants in the absence and presence of BR. Asterisks indicate significant differences based on one-way ANOVA test. *p* < 0.05. Different letters indicate significant differences, whereas the same letters indicate non-significant differences based on *post hoc* Tukey’s HSD test. *p* < 0.05. **(D)** *ABI5* expression in wild-type and *jmj30-2 jmj32-1* plants with and without ABA/BR by RT-qPCR. Asterisks indicate significant differences based on one-way ANOVA test. *p* < 0.05. Different letters indicate significant differences, whereas the same letters indicate non-significant differences based on *post hoc* Tukey’s HSD test. *p* < 0.05.

In the absence of ABA, almost all seedlings developed normally to form green cotyledons, a pair of true leaves, and copious root hairs not only in the wild type, but also in *jmj30-2 jmj32-1* double mutants (Figure 2A). In the presence of 0.3 μM ABA, approximately 80% of germinated wild-type plants failed to develop green cotyledons and the first pair of true leaves, and were arrested just after germination (Figures 2A,B). As reported previously, the *jmj30 jmj32* double mutant showed insensitive to 0.3 μM ABA treatment as compared with the wild type (ABA-treated WT and ABA-treated *jmj30 jmj32*: *p* < 0.05 by one-way ANOVA test; ABA-treated WT vs. the other samples: *p* < 0.05 by *post hoc* Tukey’s HSD test; Figures 2A,B; Wu et al., 2019a). ABA inhibits seedling establishment in the wild type, which is partially restored by BR (Figures 2A,B; Ryu et al., 2014). This restoration suggests that BR suppresses ABA signaling during the post-germination stage. Although there is no phenotype in either of the *jmj30* and *jmj32* single mutants (Supplementary Figure 3), we observed that ABA-treated double mutants and ABA/BR-treated wild type had similar seedling establishment rates, suggesting that mutations in the *JMJ* genes and exogenous BR treatment have the similar effects in terms of inhibiting ABA signaling (ABA/BR-treated WT and ABA/BR-treated *jmj30 jmj32*: *p* < 0.05 by one-way ANOVA test; Figures 2A,B). We confirmed these results by measuring the fresh weight of ABA/BR-treated wild type and ABA-treated double mutants and monitoring the expression of the ABA-regulated gene *ABI5* (Figures 2C,D). Thus, we conclude that JMJ30 and JMJ32 are redundantly required for proper BR signaling to inhibit ABA pathway.

We also examined the effects of JMJ30 and JMJ32 on ABA responses during seed germination. The timing of germination was similar in wild-type, *jmj30-2*, *jmj32-1*, and *jmj30-2 jmj32-1* seedlings following ABA and BR treatment, suggesting that ABA and BR treatment do not mediate the growth-arrest phenotype (Supplementary Figure 4).

### JMJ30 Removes H3K27me3 From the BZR1 Loci in Response to ABA

**FIGURE 3:**
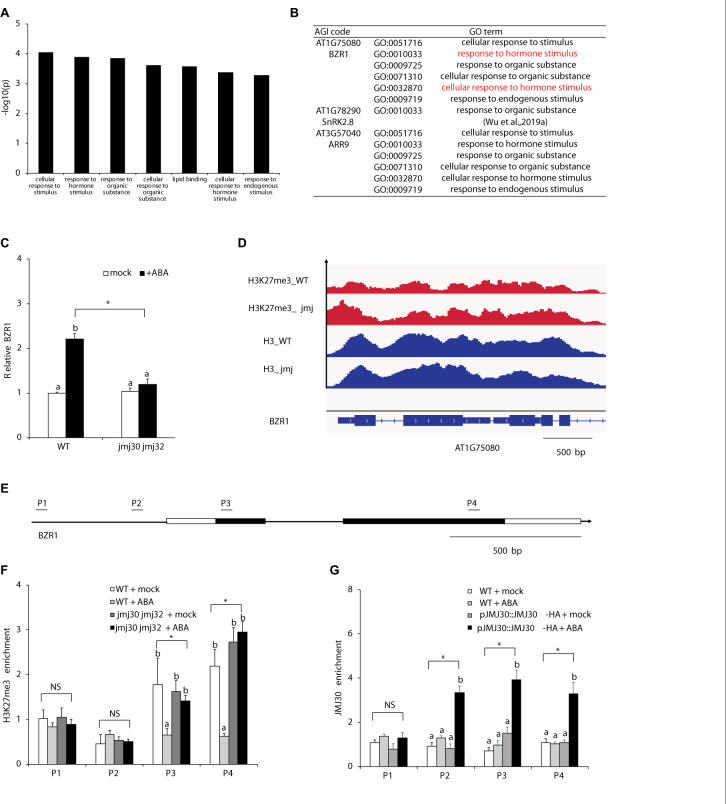
JMJ30 protein removes H3K27me3 from the *BZR1* loci in response to ABA. **(A)** AgriGO analysis revealed seven ABA- and JMJ-dependent biological processes. **(B)** The GO terms of some plant hormone response genes, such as *BZR1*, were included. **(C)** *BZR1* expression levels in wild type and *jmj30-2 jmj32-1* after ABA treatment, based on RT-qPCR. Asterisks indicate significant differences based on one-way ANOVA test. *p* < 0.05. Different letters indicate significant differences, whereas same letters indicate non-significant differences based on *post hoc* Tukey’s HSD test. *p* < 0.05. **(D)** H3K27me3 levels at the *BZR1* loci in mock-treated wild type and *jmj30-2 jmj32-1*. **(E)** PCR fragments (P) and schematic diagram of genes are shown below the ChIP data. White bars, untranslated regions; black bars, protein coding regions. **(F)** H3K27me3 ChIP at the *BZR1* loci in wild type and *jmj30-2 jmj32-1* with and without ABA. **(G)** JMJ30-HA ChIP at the *BZR1* loci in wild type and *jmj30-2 jmj32-1* with and without ABA. Values are means ± SEM from three independent experiments. Asterisks indicate significant differences based on one-way ANOVA test. *p* < 0.05. Different letters indicate significant differences, whereas same letters indicate non-significant differences based on a *post hoc* Tukey’s HSD test. *p* < 0.05. NS: non-significant.

Because JMJ mediates the crosstalk between ABA and BR, we hypothesized that JMJ30 and JMJ32 might act on BR-related downstream targets. To test this hypothesis, we re-analyzed a list of DEGs between mock- and ABA-treated *jmj30-2 jmj32-1* double mutant. Previous RNA sequencing (RNA-Seq) identified 60 DEGs in *jmj30 jmj32* with ABA treatment (Wu et al., 2019a). The top seven Gene Ontology (GO) terms contain two hormone-related descriptions: “Response to hormone stimulus” and “Cellular response to hormone stimulus”. The *BZR1* gene, a master transcription factor of BR signaling was found in six out of these seven GO term categories (Figures 3A,B and Supplementary Table 2; Ryu et al., 2007). RT-qPCR analysis confirmed that *BZR1* expression was upregulated after ABA treatment in wild type, but not in mock- and ABA-treated *jmj30 jmj32* mutants (Figure 3C). Thus, we focused on *BZR1* regulation by JMJ30 and JMJ32.

To test if JMJ30 and JMJ32 upregulate *BZR1* in response to ABA through histone demethylation, we first measured the H3K27me3 levels in the *BZR1* locus. In a published genome-wide ChIP-seq dataset, the gene body of the *BZR1* was covered with H3K27me3 repressive marks in the absence of hormones, in both wild type and *jmj30 jmj32 ref6 elf6* mutants (Figure 3D; Yamaguchi et al., 2020). H3 levels were also similar in these samples (Figure 3D). To confirm the effects of ABA treatment on H3K27me3 levels, we performed a ChIP assay followed by quantitative PCR (ChIP-qPCR). We detected H3K27me3 marks in the gene body at the *BZR1* locus (fragments P3 and P4) in mock-treated wild type and *jmj30-2 jmj32-1* mutants (Figure 3E). Upon ABA treatment, these marks decreased in the wild type, but not in *jmj30-2 jmj32-1* mutants (P3 and P4: *p* < 0.05 by one-way ANOVA test; ABA-treated wild type vs. the other samples: *p* < 0.05 by *post hoc* Tukey’s HSD; Figure 3F).

To further test if JMJ30 directly regulates *BZR1*, we carried out ChIP-qPCR with a biologically active JMJ30 protein tagged with the human influenza hemagglutinin (HA) epitope-the *pJMJ30:JMJ30-HA* line. Consistent with the H3K27me3 ChIP result, we detected JMJ30-HA association within the promoter and gene body of *BZR1* only in the presence of ABA (P2, P3, and P4: *p* < 0.05 by one-way ANOVA test; ABA-treated JMJ30-HA vs. the other samples: *p* < 0.05 by *post hoc* Tukey’s HSD test; Figure 3G). These results indicate that JMJ30 removes H3K27me3 at the *BZR1* locus in the presence of ABA.

### Reduced JMJ Activity and *BZR1* Overexpression Synergistically Promote Plant Growth and Development in the Presence of ABA

To further understand the relationship between JMJs and BZR1, we examined how ectopic expression of BZR1 could affect the ABA insensitivity of *jmj30-2 jmj32-1* double mutants. We overexpressed *BZR1* under the control of the Cauliflower mosaic virus *35S* promoter (*BZR1 ox*) in the double mutants. Of the eight independent lines, *jmj30-2 jmj32-1 BZR1 ox* line 1 (L1) had a similar expression level to the ABA-treated wild type (Supplementary Figure 5). We also identified six *jmj30-2 jmj32-1 BZR1 ox* lines that possessed ∼20-fold (L2-L7) to over 40-fold (L8) increases in the amount of *BZR1* transcripts relative to that of ABA-treated wild-type seedlings (Supplementary Figure 5). Consistent with previous findings (Yang et al., 2016), *BZR1 ox* L1 and L8 lines displayed strong and weak germination defects, respectively (Supplementary Figure 6). We used the L1 and L8 lines for further phenotypic analyses.

**FIGURE 4:**
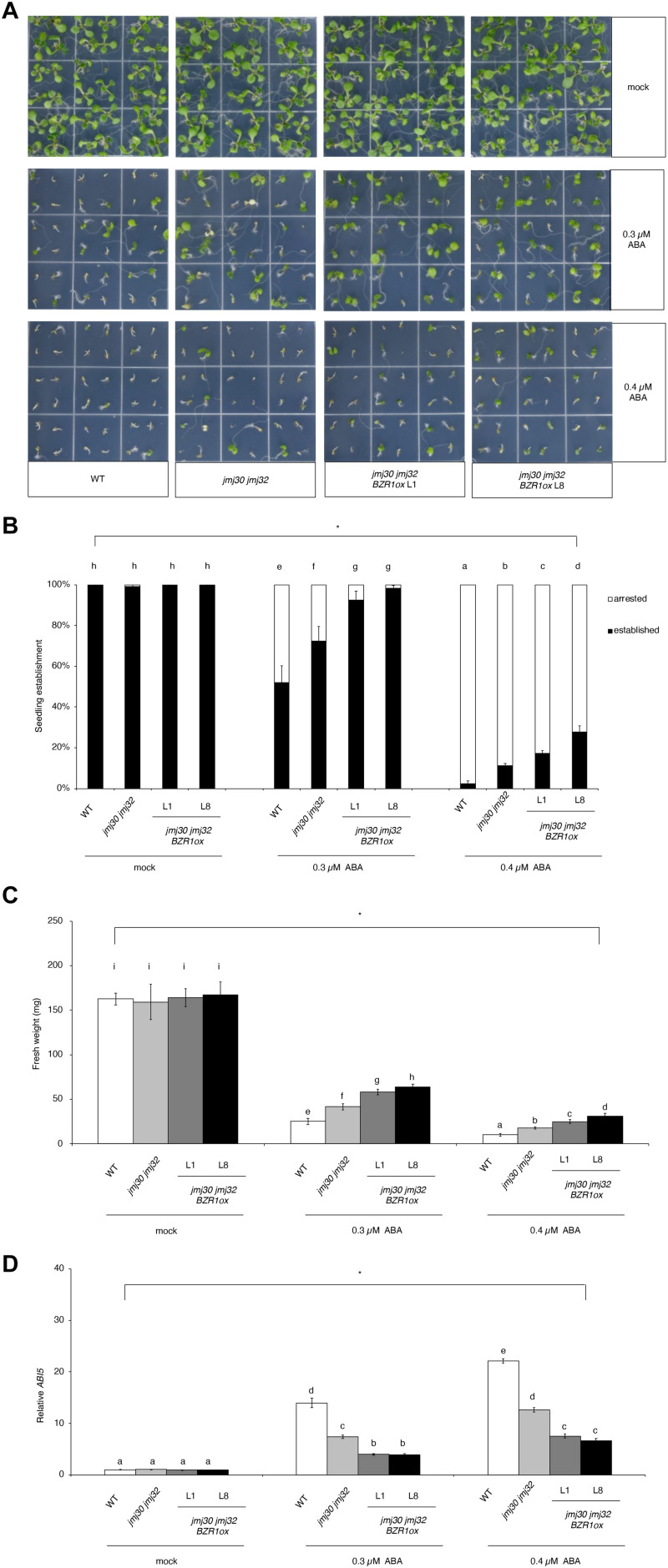
Overexpression of *BZR1* in *jmj30 jmj32* attenuates ABA sensitivity. **(A)** Representative images of wild-type, *jmj30-2 jmj32-1*, *jmj30-2 jmj32-1 BZR1ox* L1, and *jmj30-2 jmj32-1 BZR1ox* L8 seedlings grown for seven days on 1/2 MS medium (-sucrose) with 0, 0.3, or 0.4 μM ABA. **(B)** Percentage of established and arrested seedlings in wild-type, *jmj30-2 jmj32-1*, *jmj30-2 jmj32-1 BZR1ox* L1, and *jmj30-2 jmj32-1 BZR1ox* L8 plants in the absence and presence of ABA. Asterisks indicate significant differences based on one-way ANOVA test. *p* < 0.05. Different letters indicate significant differences based on a *post hoc* Tukey’s HSD test. *p* < 0.01. NS: non-significant. **(C)** Measurement of the fresh weight of wild-type, *jmj30-2 jmj32-1*, *jmj30-2 jmj32-1 BZR1ox* L1, and *jmj30-2 jmj32-1 BZR1ox* L8 seedlings in the absence and presence of ABA. Asterisks indicate significant differences Continued based on a one-way ANOVA test. *p* < 0.05. Different letters indicate significant differences based on a *post hoc* Tukey’s HSD test. *p* < 0.01. NS: non-significant. Values in panels **(B,C)** represent means ± SEM of 36 plants. **(D)** *ABI5* expression in wild-type, *jmj30-2 jmj32-1*, *jmj30-2 jmj32-1 BZR1ox* L1, and *jmj30-2 jmj32-1 BZR1ox* L8 seedlings with and without ABA/BR by RT-qPCR. Asterisks indicate significant differences based on one-way ANOVA test. *p* < 0.05. Different letters indicate significant differences, whereas the same letters indicate non-significant differences based on *post hoc* Tukey’s HSD test. *p* < 0.05.

In the absence of ABA, wild type, *jmj30-2 jmj32-1*, and *jmj30-2 jmj32-1 BZR1 ox* L1 and L8 plants grew normally after germination (Figure 4A). The double mutants had higher seedling establishment rates after 0.3 μM ABA treatment, compared with wild type, as reported previously (Figures 4A,B; Wu et al., 2019a). We observed further increasing seedling establishment rates in *jmj30 jmj32* when we overexpress the *BZR1* gene (*p* < 0.01 by one-way ANOVA test; 0.3 or 0.4 μM ABA-treated *jmj30 jmj32* vs. the other samples: *p* < 0.01 by *post hoc* Tukey’s HSD; Figure 4B). We obtained similar results when we measured fresh weights (*p* < 0.01 by one-way ANOVA test; 0.3 μM or 0.4 μM ABA-treated *jmj30 jmj32* vs. the other samples: *p* < 0.01 by *post hoc* Tukey’s HSD test; Figure 4C) and *ABI5* gene expression (Figure 4D). Thus, we conclude that reduced JMJ activity (and hence reduced ABA/SnRK2.8-mediated stress response) and *BZR1* overexpression strongly promote plant growth and development in the presence of ABA.

### JMJs Control Cellular Homeostasis During Post-germination Stage by Increasing *BZR1* Expression

**FIGURE 5:**
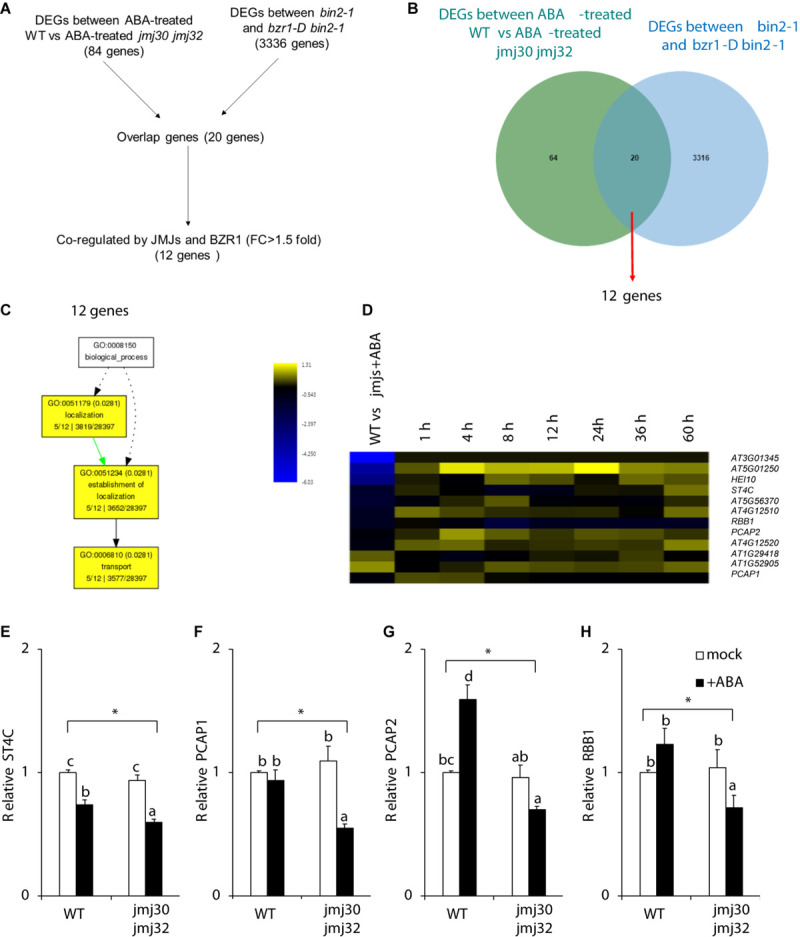
JMJs and BZR1 coordinately regulate transport-related genes. **(A)** The flowchart of candidate genes selection. **(B)** Venn diagram showing the number of genes differentially expressed in ABA-treated wild type (WT) and *jmj30-2 jmj32-1*, and the number of genes differentially expressed in *bin2-1* and *bzr1-D bin2-1* plants. **(C)** GO analysis among 12 co-regulated genes. **(D)** Heatmap displaying the log_2_FC (fold change) of the 12 candidates based on two transcriptome datasets. **(E–H)** Representative genes in wild-type and *jmj30-2 jmj32-1* plants with and without ABA by RT-qPCR: **(E)** *ST4C*, **(F)** *PCAP1*, **(G)** *PCAP2*, and **(H)** *RBB1*. Asterisks indicate significant differences based on a one-way ANOVA test. *p* < 0.05. Different letters indicate significant differences, whereas same letters indicate non-significant differences based on a *post hoc* Tukey’s HSD test. *p* < 0.05.

Our results suggest that JMJs and BZR1 modulate the same pathway. We hypothesized that JMJs and BZR1 must have common downstream targets if they control ABA-dependent growth arrest. To identify these genes, we conducted computational analysis using public RNA-seq datasets (Yang et al., 2016; Wu et al., 2019a). Although the plant growth conditions of those datasets were not identical, a third transcriptome dataset (Klepikova et al., 2016) supports the conclusion that some genes are expressed at the growth stage investigated in our study (Supplementary Figure 7). Therefore, we made use of publicly available RNA-seq datasets and identified 20 genes for which expression levels were co-regulated by *JMJs* and *BZR1* (Figures 5A,B). Among these, 12 genes were co-regulated by JMJs and BZR1 and possessed >1.5-fold differences relative to controls (Figures 5A,B and Supplementary Table 3). To examine the likely functions of these 12 genes, we tested for GO term enrichment analysis using agriGO. These 12 genes were categorized into “localization,” “establishment of localization,” and “transport” (Figure 5C).

Figure 5D shows a clustered heatmap of the expression changes observed in two datasets (Song et al., 2016; Wu et al., 2019a). Eight genes were upregulated and four genes were downregulated in wild type following ABA treatment (Figure 5D and Supplementary Table 4). Most of the eight genes co-regulated by JMJs and ABA had reached their highest expression levels at 4 h after ABA treatment. These eight genes contain three transport proteins: a regulator of vacuole morphology, *REGULATOR OF BULB BIOGENESIS1* (*RBB1*); and two lipid-transfer proteins, *PLASMA-MEMBRANE ASSOCIATED CATION-BINDING PROTEIN1* (*PCAP1*) and *PCAP2* Each of these plays a unique role in transport (Figure 5D). A cytokinin responsive regulator, *SULFOTRANSFERASE 4C* (*ST4C*) was identified.

To confirm the RNA-seq results, we examined the expression of these downstream genes in the presence and absence of ABA during post-germination stage. We chose the above four genes because *RBB1*, *PCAP1*, and *PCAP2* are linked to transport- and establishment of localization-related GO terms, and *ST4C* is involved in cytokinin response (Lee et al., 2007; Kato et al., 2010; Han et al., 2015; Nagata et al., 2016). Without ABA treatments, there were no expression differences in these genes between wild type and double mutants (Figures 5E–H). In the presence of ABA, the expression of all four genes was significantly higher in the wild type than in the double mutants (*p* < 0.05 by one-way ANOVA test; WT + ABA vs. the other samples: *p* < 0.05 by *post hoc* Tukey’s HSD; Figures 5E–H). Thus, JMJs and BZR1 may co-regulate genes that are involved in cellular homeostasis during the post-germination stage.

### BZR1 Also Regulates ABA/BR Response in a JMJ-Independent Pathway

**FIGURE 6:**
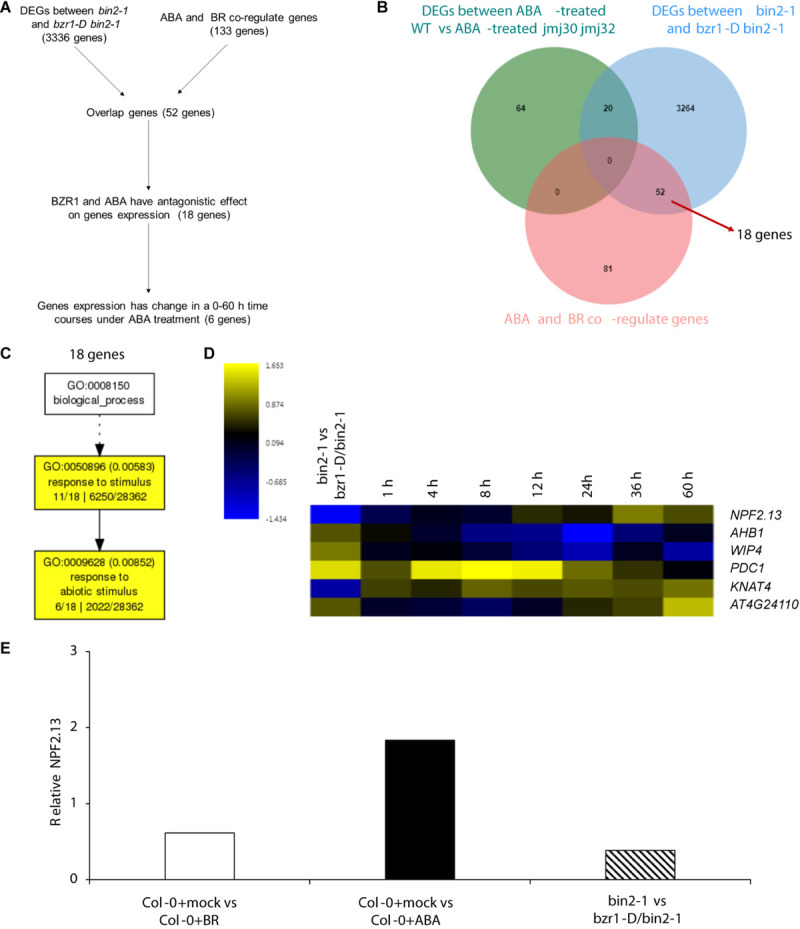
BZR1 regulate ABA/BR responsive genes independently. **(A)** The flowchart of candidate genes selection. **(B)** Venn diagram showing the number of genes differentially expressed in ABA-treated wild-type (WT) and *jmj30-2 jmj32-1* plants, the number of genes differentially expressed in *bin2-1* and *bzr1-D bin2-1*plants and the number of ABA/BR co-regulated genes. **(C)** The GO analysis among 18 BZR1 and ABA/BR co-regulated genes. **(D)** Heatmap displaying the log_2_FC (fold change) of the 18 candidates based on two transcriptome datasets. **(E)** Relative expression of *NPF2.13* by RNA-seq. Y-axis represents FC. *p* < 0.05.

Because strong *BZR1ox* together with the mutations in the *JMJ* genes almost fully inhibited ABA signaling during post-germination stage, we hypothesized that BZR1 may function independently of JMJ-mediated histone modification. To test this hypothesis, we first screened JMJ-independent BZR1 downstream genes by comparing DEGs between ABA-treated WT vs ABA-treated *jmj30 jmj32* with two transcriptome datasets of BZR1 targets and ABA-/BR co-regulated genes (Wang et al., 2018a). We identified 3316 genes (Figures 6A,B), of which 52 genes were affected by exogenous ABA/BR treatment without JMJs function. Because *bzr1-D*, which is a gain-of-function mutant of *BZR1*, exhibits constitutive BR responses, gene regulation in *bzr1-D* and ABA-treated plants are predicted to work in opposite directions. Indeed, we observed this opposite expression pattern for 18 out of 52 genes (Figures 6A,B and Supplementary Table 5). These were involved in “response to stimuli” and “response to abiotic stimuli” based on GO term analysis (Figure 6C).

Of the 18 genes, expression levels of six were temporally regulated during post-germination stage (Song et al., 2016). Among those six genes, two genes*—PYRUVATE DECARBOXYLASE 1* (*PDC1*) and *KNOTTED1-LIKE HOMEOBOX GENE 4* (*KNAT4*)—reached their highest expression levels eight hours after ABA treatment, the expression of *AT4G24110* and *NRT1/PTR FAMILY 2.13* (*NPE2.13*) reached their highest expression levels 60 hours after ABA treatment. The *PHYTOGLOBIN 1* (*AHB1*) and *WIP DOMAIN PROTEIN 4* (*WIP4*) expression were inhibit by ABA (Figure 6D). Three of these genes—*PDC1, AT4G24110*, and *AHB1*—are linked to “cellular response to hypoxia” (Supplementary Table 6). *NPE2.13* was regulated in the same direction as *bzr1-D* in BR-treated plants and in the opposite direction in ABA-treated plants (Figure 6E). These results suggest that BZR1 also regulates ABA/BR-responses during the post-germination stage via a JMJ-independent pathway.

## Discussion

### JMJs Play an Important Role in Integrating BR and ABA Crosstalk

**FIGURE 7:**
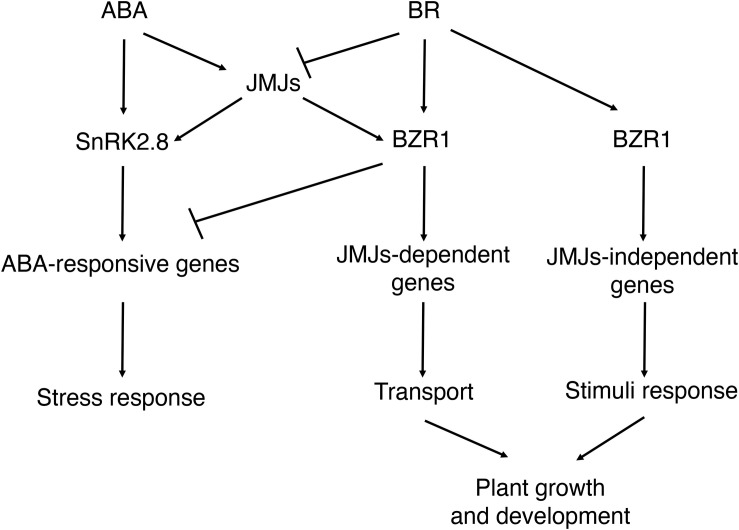
Proposed models for the interaction between ABA and BR during post-germination stage. ABA inhibits the later growth of the seedling. BR promotes the later growth of the seedling. JMJ-dependent and JMJ-independent pathway modulate growth arrest upon perception of ABA and BR signals post-germination.

In this study, we found that histone demethylases JMJ30 and JMJ32 integrate inputs from ABA and BR during post-germination stage (Figure 7). Subsequently, JMJ30 and JMJ32 control the expression two downstream targets, *SnRK2.8* and *BZR1* depending on the hormonal inputs. Since those two genes have opposite functions during post-germination stage, JMJ30 and JMJ32 could determine the balance between stress response and growth by regulating those targets. Furthermore, this crosstalk is regulated by both JMJ-dependent and JMJ-independent pathways.

Our study provides genetic and biochemical evidence to support the role of JMJs in integrating ABA- and BR-signaling. ABA upregulates JMJ30 and BR downregulates it (Wu et al., 2019a, b). BR-mediated repression of ABA-inducible genes was absent in *jmj30 jmj32* mutants (Supplementary Figure 2). Exogenous BR treatment and the mutations in the *JMJ30* and *JMJ32* genes have the same effects for inhibiting ABA signaling (Figure 2). Although we previously revealed that ABI3 activated *JMJ30* expression upon ABA treatment, transcription factors that repress *JMJ30* expression in response to BR are not identified, yet. Based on the publicly available ChIP datasets, BZR1 binds to *JMJ30*. Although the ChIP experiment was conducted in a different plant growth stage, BZR1 could be a regulator of *JMJ30*. Identification and/or verification of these transcription factors will elucidate how this integration is regulated. Antagonistic relationship on gene expression is often regulated by competitive binding at common regulatory regions. Interestingly, the *JMJ30* promoter region around an ABI3 binding site is evolutionally conserved in *Brassicaceae* spices (Wu et al., 2019a). Thus, such mechanism might also exist for regulating *JMJ30* expression by ABA and BR.

### JMJs Directly Regulate Both BR- and ABA-Related Downstream Targets

We have identified genes critical to ABA-mediated growth arrest, which are regulated through JMJ-dependent histone modification. These genes were linked to many biological processes including hormonal control. *SnRK2.8* acts downstream of JMJ30 in ABA-mediated growth arrest (Wu et al., 2019a). In this study, we identified the master regulator of BR signaling, *BZR1*, as another JMJ30 target (Figure 3Supplementary Table 2). We have shown that *BZR1* is a direct target of JMJs in the presence of ABA (Figure 3). Based on the previous *in vitro* demethylase assay (Gan et al., 2014), JMJ30 specifically demethylates oligonucleosomes at H3K27me2/3, but not H3K9me2/3. Although H3K9ac changes were reported in *elf6 ref6* mutants by the previous publication (Yu et al., 2008), JMJ30 is likely to demethylate H3K27me3. Indeed, ABA-dependent removal of H3K27me3 at *BZR1* was regulated via JMJ30 and JMJ32. Thus, JMJ30 and JMJ32 control the ABA and BR signal transduction pathways by regulating distinct downstream targets to balance growth and stress responses through H3K27me3 removal (Figure 7). Overall, H3K27 demethylases tend to have redundant roles during plant development and stress responses (Lu et al., 2011; Gan et al., 2014; Zheng et al., 2019; Yamaguchi et al., 2020). Unlike JMJ30 and JMJ32, ELF6, and REF6 could also control H3K9me3 in a direct way by unknown mechanisms (Yu et al., 2008). Further analysis using multiple mutants is required to figure out their functional differences in response to ABA. Our genetic analysis revealed that reduced JMJ30 and JMJ32 activities (and hence reduced ABA-JMJ30-SnRK2.8 pathway) and *BZR1* overexpression synergistically promote plant growth and development in the presence of ABA (Figure 4). Furthermore, our results also support that BZR1 represses ABA-responsive gene expression as reported previously (Yang et al., 2016). Not only JMJ, but also BZR1 control ABA-BR crosstalk (Figure 7).

### BZR1 Regulates ABA-Mediated Growth Arrest in JMJ-Dependent and JMJ-Independent Pathways

Phenotypic analysis using *BZR1ox* and *jmj30 jmj32* double mutants suggested the existence of two pathways involved in ABA-dependent growth arrest: JMJ-dependent and JMJ-independent. We identified genes involved in the two distinct pathways using public transcriptome datasets.

The genes that act in the JMJ-BZR1 pathway are membrane proteins linked to transport (Figure 5 and Supplementary Table 3). The four genes transcriptionally regulated by JMJ and BZR1 include *RBB1*, *PCAP1*, *PCAP2*, and *ST4C*. The membrane protein RBB1 controls vacuole bulb formation (Han et al., 2015). Bulbs are located in the epidermis of the root and may act as membrane reservoirs for cell expansion (Uemura et al., 2002). PCAP2 is a plasma membrane-associated Ca^2+^-binding protein (Zhu et al., 2013), which acts in ABA response and in the crosstalk between ABA and salicylic acid (SA) during root growth (Wang et al., 2018b, c). We hypothesize that JMJ and BZR1 co-regulate *PCAP2* expression to control the crosstalk between ABA and BR signals. *PCAP1*, a homolog of *PCAP2*, is also a plasma membrane-associated Ca^2+^-binding protein (Tanaka-Takada et al., 2019). Although functional characterization using both homologs has not been conducted yet, PCAP1 may share functions with *PCAP2*. The cytokinin responsive regulator, *ST4C*, is a common target of JMJ and BZR1 (Figure 5E). Cytokinin signaling antagonizes ABA-mediated inhibition of seedling growth (Huang et al., 2018). Overall, these four genes may control root growth in the JMJ-BZR1-dependent pathway.

On the other hand, some genes that are linked to stress response function in the BZR1 pathway independently of JMJs (Figure 6 and Supplementary Table 3). These genes are enriched for the GO terms “response to stimuli” and “response to abiotic stimuli” (Figure 6C). Four well-characterized proteins were involved in BZR1 and JMJ-independent pathways (Figure 6D). Two candidates, *PDC1* and *AHB1*, respond to hypoxic stress. *PDC1* enhances plant survival under low oxygen stress (Ismond et al., 2003) and *AHB1* reduces NO emission to help plant escape from hypoxic stress (Perazzolli et al., 2004). Thus, BZR1 may affect gene expression in the hypoxic response pathway to integrate ABA and BR signals. The third candidate, *NPF2.13*, is responsible for normal nitrogen cycling under low nitrogen stress (Nour-Eldin et al., 2012; Leran et al., 2014). The fourth candidate, *KNAT4*, was identified as a BZR1 target. Its expression in the elongation zone of the root is triggered by cytokinin and light (Truernit et al., 2006). Therefore, JMJ-independent genes that are modulated by BZR1 may play key roles in various stress responses.

Although we have identified two different pathways, we still don’t know their spatial and temporal domains. JMJ30 accumulated mainly in the root meristematic and maturation zones (Figure 1), whereas BZR1 is expressed in the root elongation and maturation zones. Thus, the JMJ-BZR1 pathway might function in the maturation zone of root, where those genes are co-expressed. In contrast, the JMJ-independent BZR1 pathway may function in the root elongation zone. Further investigation is required to specify the exact function of these pathways.

## Data Availability Statement

The original contributions presented in the study are included in the article/Supplementary Material, further inquiries can be directed to the corresponding authors.

## Author Contributions

JW, NY, and TI: conceptualization. JW and NY: data curation, formal analysis, investigation, software, visualization, and writing – original draft. MY, NY, and TI: funding acquisition. NY: project administration and validation. NY and TI: supervision. All authors: review and editing.

## Conflict of Interest

The authors declare that the research was conducted in the absence of any commercial or financial relationships that could be construed as a potential conflict of interest.

## Footnotes

1 http://jvenn.toulouse.inra.fr/app/example.html

2 http://mev.tm4.org/

## Supplementary Material

Supplementary Table 1 Primers used in this study.

Supplementary Table 2 DEGs are from seven GO term.

Supplementary Table 3 12DEGs co-regulated by JMJs and BZR1.

Supplementary Table 4 12 candidates expression change under ABA-treated time courses.

Supplementary Table 5 18 genes expression were regulated by BZR1 and ABA/BR treatment.

Supplementary Table 6 Six candidates expression change under ABA-treated time courses.
