# Supplementary material for: Histone Demethylases Coordinate the Antagonistic Interaction Between Abscisic Acid and Brassinosteroid Signaling in Arabidopsis
Source: Front Plant Sci. 2020 Nov 25;11:596835. doi: 10.3389/fpls.2020.596835 (PMC7724051; doi:10.3389/fpls.2020.596835)
Supplement: Supplementary file 7 [file Data_Sheet_1.PDF]

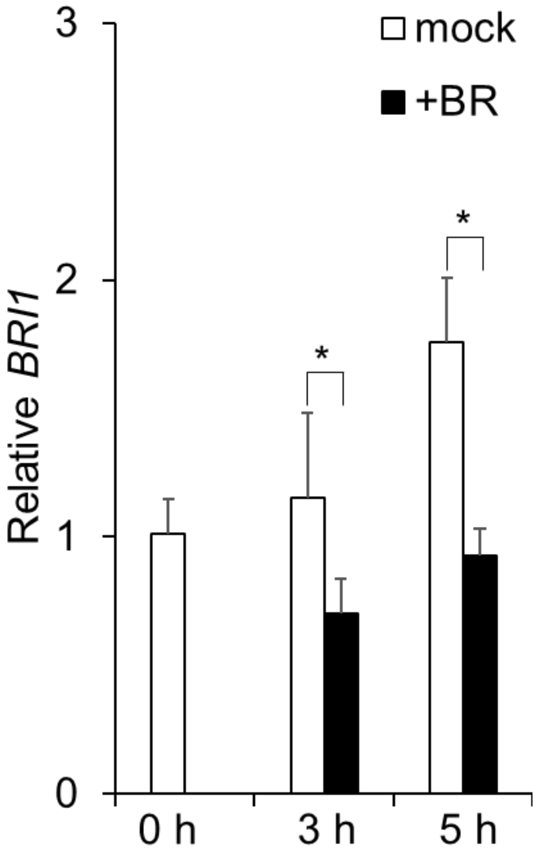

Figure S1. *BRI1* expression following BR treatment during post-germination.

BRI1 in wild-type seedlings in response to BR during germination. Results are from three independent experiments. Values represent mean ± SEM. Asterisks indicate significant differences between mock- and ABA-treated plants based on two-tailed Student's *t*-test.

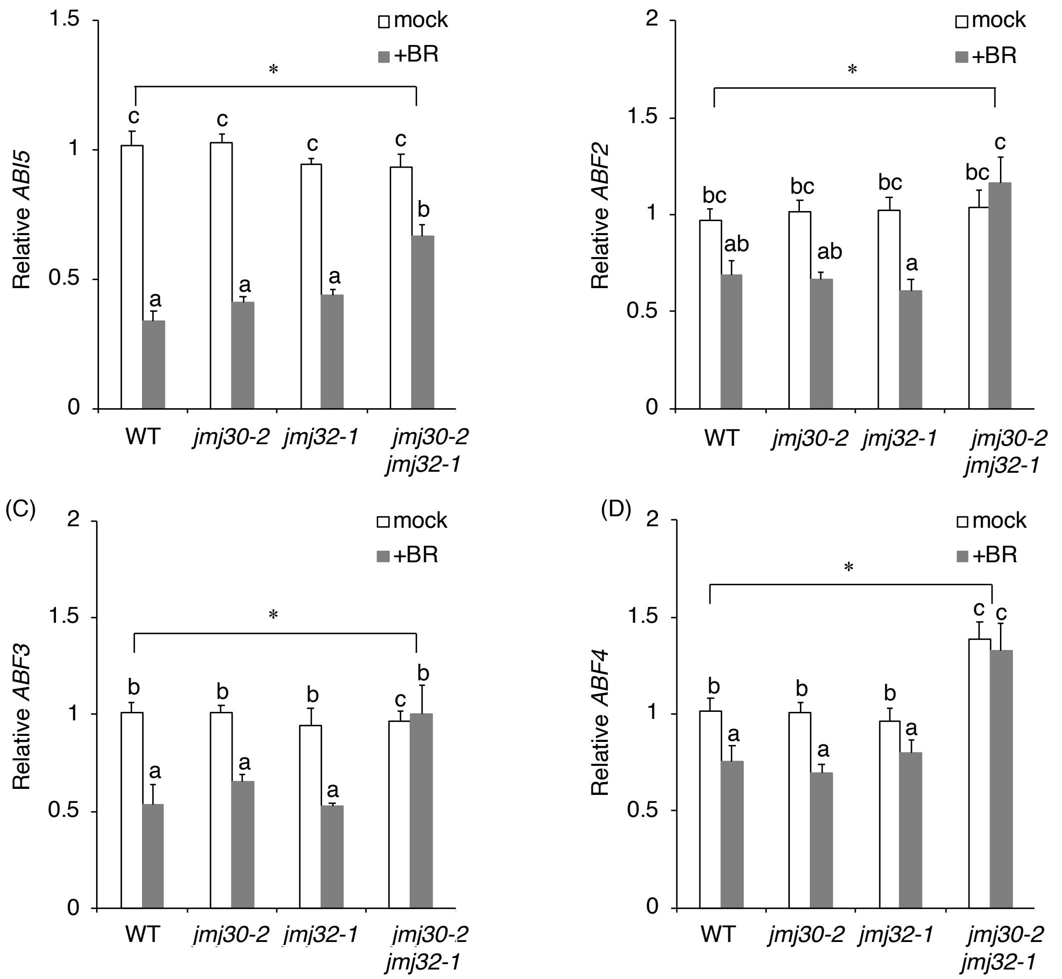

Figure S2. JMJs repress the expression of ABA-responsive genes under BR treatment.

(A–D) Expression of ABA-responsive genes in BR-treated wild-type plants compared with BR-treated *jmj30-2*, *jmj32-1*, and *jmj30-2 jmj32-1* mutants by RT-qPCR. **(A)** *ABI5*. **(B)** *ABF2*. **(C)** *ABF3*. **(D)** *ABF4*. Values are mean ± SEM from three independent experiments. Different letters indicate significant differences based on post hoc Tukey's HSD test. $P < 0.05$. Asterisks indicate significant differences based on one-way ANOVA test.

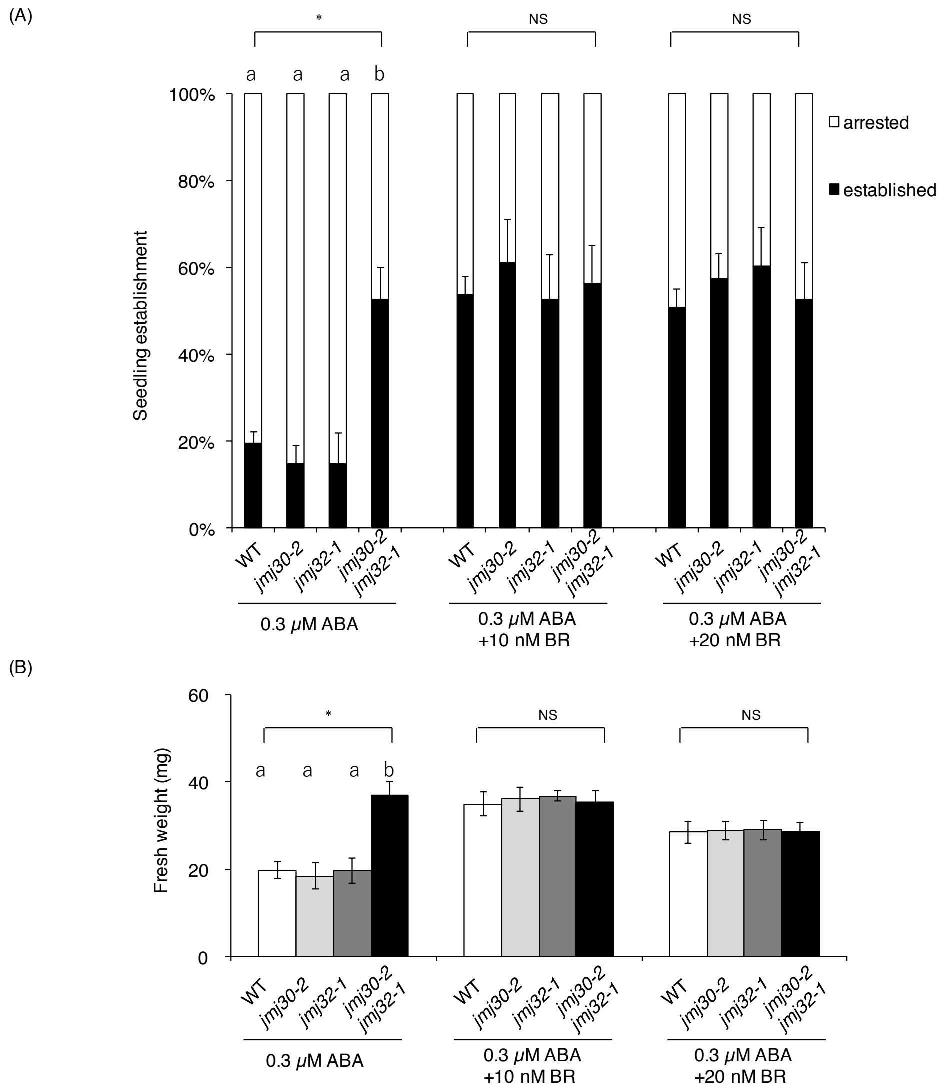

Figure S3. Seedling-arrest phenotype in *jmj* mutants during the post-germination stage.

(A) Percentages of established seedlings in ABA-treated wild-type, *jmj30-2*, *jmj32-1*, and *jmj30-2 jmj32-1* plants in the absence and presence of BR. Asterisks indicate significant differences based on one-way ANOVA test. $p < 0.05$. Different letters indicate significant differences, whereas the same letters indicate nonsignificant differences based on post-hoc Tukey's HSD test. $p < 0.05$. **(B)** Measurement of fresh weight in ABA-treated wild-type, *jmj30-2*, *jmj32-1*, and *jmj30-2 jmj32-1* plants in the absence and presence of BR. Asterisks indicate significant differences based on one-way ANOVA test. $p < 0.05$. Different letters indicate significant differences, whereas the same letters indicate nonsignificant differences based on post-hoc Tukey's HSD

test. $p < 0.05$. For the wild type and *jmj30 jmj32* double mutant, the same data were used for Fig. 2 .

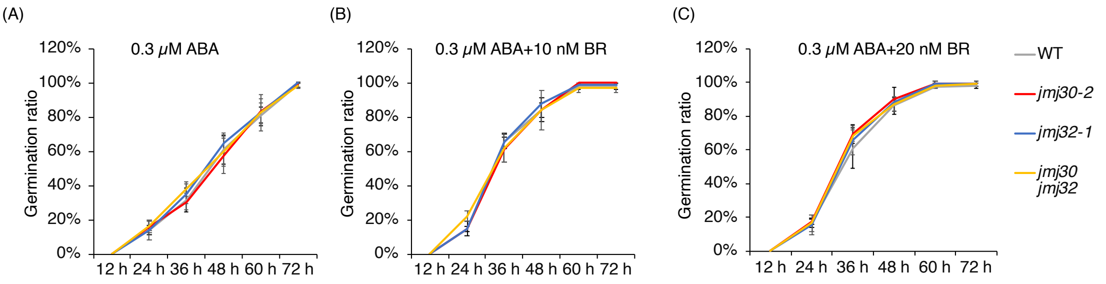

Figure S4. Germination rates of the wild type and *jmj30*, *jmj32*, and *jmj30 jmj32* mutants with ABA and BR treatment.

(A–C) Rates of seed germination on 1/2 MS medium (−sucrose) plates in the absence of BR **(A)** and in the presence of 10 nM **(B)** and 20 nM **(C)** BR. Values represent mean ± SD.

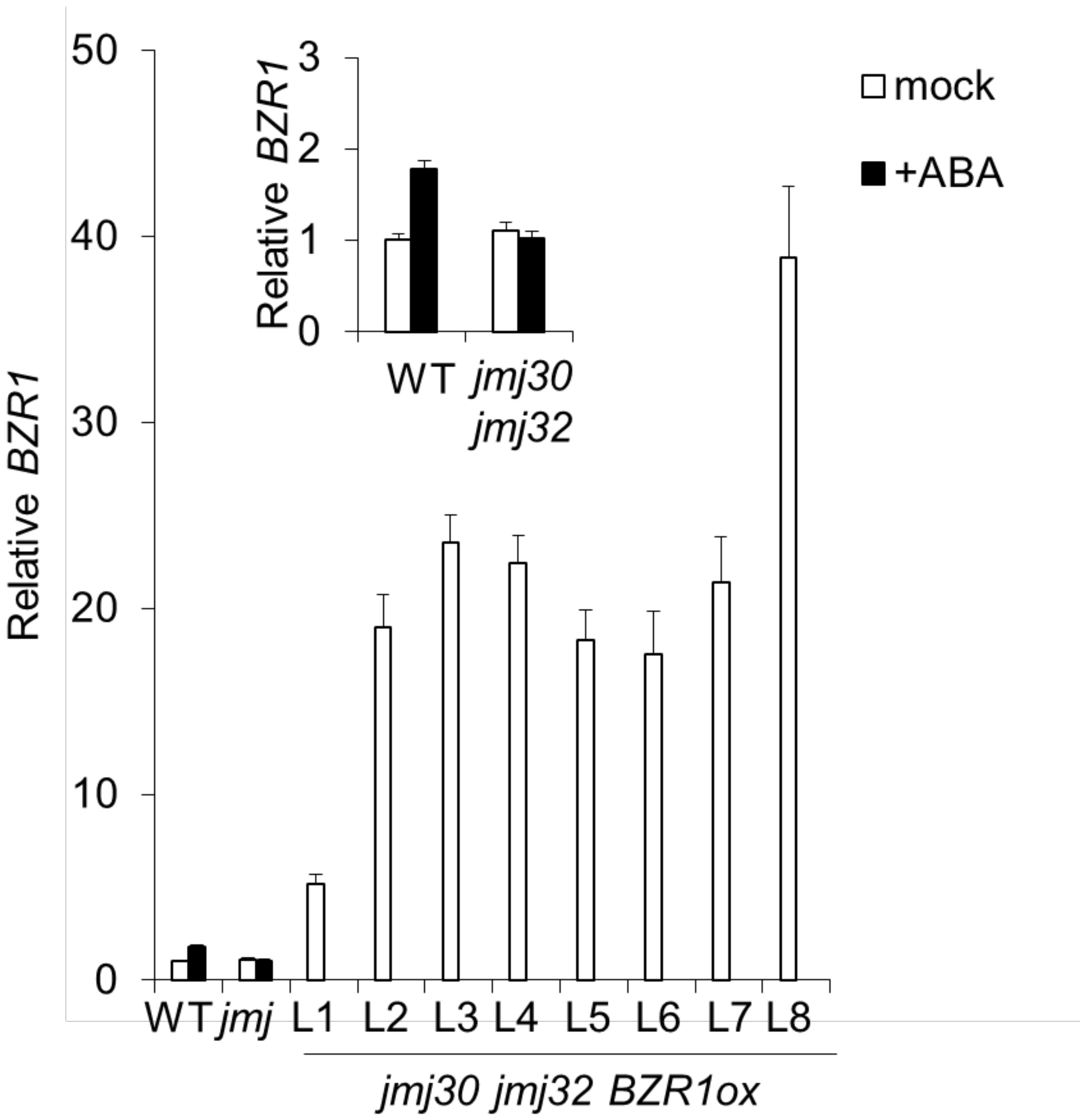

Figure S5. ***BZR1*** **expression in** ***jmj30 jmj32*** **double mutants.**

Expression of *BZR1* in eight independent *jmj30-2 jmj32-1 BZR1ox* lines. Values represent mean ± SEM.

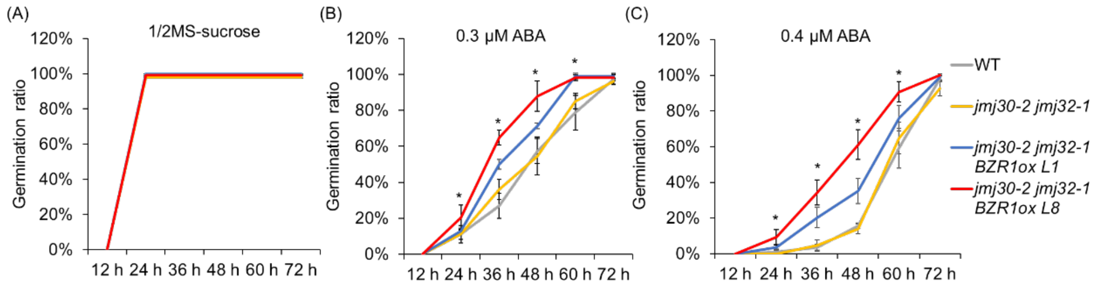

Figure S6. Germination rates between wild type, *jmj30 jmj32* and *jmj30 jmj32 BZR1ox* lines with and without ABA.

(A-C) Rates of seed germination on 1/2 MS medium (-sucrose) plates in the absence **(A)** and in the presence of 0.3 µM **(B)** and 0.4 µM **(C)** ABA. Values represent mean ± SD. Asterisks indicate statistical significance based on Chi-Squared test.

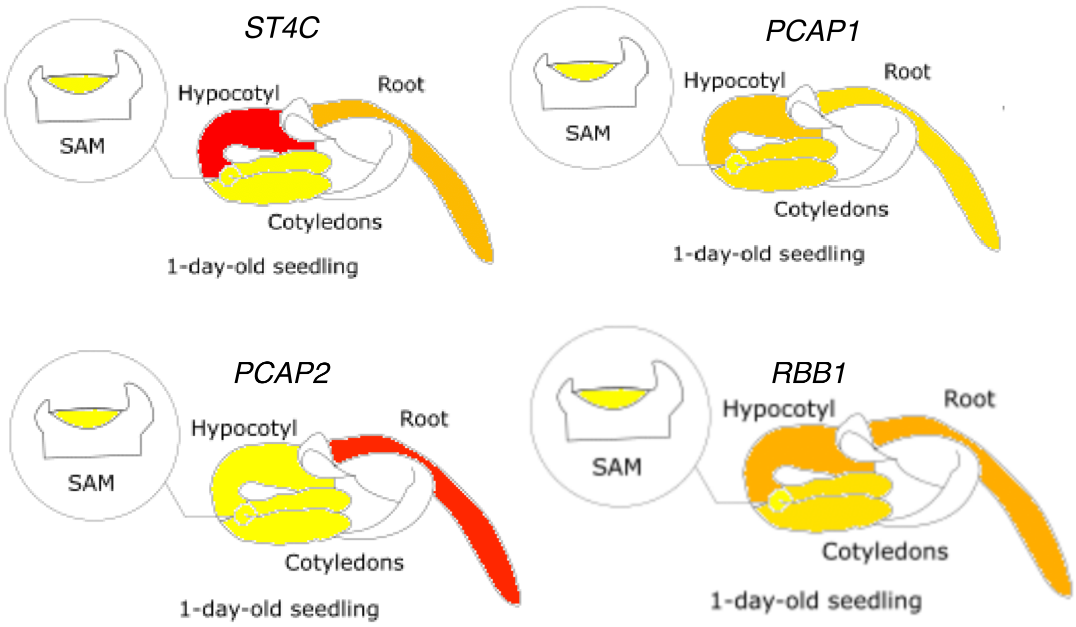

Figure S7. Expression pattern of JMJ-regulated genes during the post-germination stage.

Expression pattern of *ST4C*, *PCAP1*, *PCAP2*, and *RBB1* from the TAIR website.
